# Expression of Concern: Novel fatty chain-modified GLP-1R G-protein biased agonist exerts prolonged anti-diabetic effects through targeting receptor binding sites

**DOI:** 10.1039/d2ra90118h

**Published:** 2022-11-22

**Authors:** Maorong Wang, Ping Yao, Minpeng Gao, Jian Jin, Yerong Yu

**Affiliations:** Department of Endocrinology, West China Hospital, Sichuan University Chengdu 610041 Sichuan China 1100312222@vip.jiangnan.edu.cn yerongyu@scu.cn; Department of Endocrinology, Affiliated Hospital of Hubei University for Nationalities Enshi 445000 Hubei China; China Pharmaceutical University Nanjing Jiangsu P. R. China; Jiangnan University Wuxi Jiangsu P. R. China

## Abstract

Expression of Concern for ‘Novel fatty chain-modified GLP-1R G-protein biased agonist exerts prolonged anti-diabetic effects through targeting receptor binding sites’ by Maorong Wang *et al.*, *RSC Adv.*, 2020, **10**, 8044–8053, https://doi.org/10.1039/C9RA10593J.

The following article ‘Novel fatty chain-modified GLP-1R G-protein biased agonist exerts prolonged anti-diabetic effects through targeting receptor binding sites’ has been published in *RSC Advances*.

The Royal Society of Chemistry was contacted by a reader who raised concerns about scientific errors in this article, and that some of the content may have been reproduced without appropriate acknowledgement.

The authors were contacted for comment but have not responded to these concerns.

*RSC Advances* is publishing this Expression of Concern to alert readers to the concerns raised. An Expression of Concern will continue to be associated with the article until we receive conclusive evidence regarding the reliability of the reported data.

Laura Fisher

15/11/2022

Executive Editor, *RSC Advances*

## Supplementary Material

